# Hydrogen Sulfide (H_2_S- or H_2_S_n_-Polysulfides) in Synaptic Plasticity: Modulation of NMDA Receptors and Neurotransmitter Release in Learning and Memory

**DOI:** 10.3390/ijms26073131

**Published:** 2025-03-28

**Authors:** Constantin Munteanu, Anca Irina Galaction, Gelu Onose, Marius Turnea, Mariana Rotariu

**Affiliations:** 1 Department of Biomedical Sciences, Faculty of Medical Bioengineering, University of Medicine and Pharmacy “Grigore T. Popa”, 700454 Iasi, Romania; anca.galaction@umfiasi.ro (A.I.G.); mariana.rotariu@umfiasi.ro (M.R.); 2 Neuromuscular Rehabilitation Clinic Division, Clinical Emergency Hospital “Bagdasar-Arseni”, 041915 Bucharest, Romania; gelu.onose@umfcd.ro; 3 Faculty of Medicine, University of Medicine and Pharmacy “Carol Davila”, 020022 Bucharest, Romania

**Keywords:** hydrogen sulfide, NMDA receptor, synaptic plasticity, neurotransmitter release, cognitive function

## Abstract

Hydrogen sulfide (H_2_S) has emerged as a pivotal gaseous transmitter in the central nervous system, influencing synaptic plasticity, learning, and memory by modulating various molecular pathways. This review examines recent evidence regarding how H_2_S regulates NMDA receptor function and neurotransmitter release in neuronal circuits. By synthesizing findings from animal and cellular models, we investigate the impacts of enzymatic H_2_S production and exogenous H_2_S on excitatory synaptic currents, long-term potentiation, and intracellular calcium signaling. Data suggest that H_2_S interacts directly with NMDA receptor subunits, altering receptor function and modulating neuronal excitability. Simultaneously, H_2_S promotes the release of neurotransmitters such as glutamate and GABA, shaping synaptic dynamics and plasticity. Furthermore, reports indicate that disruptions in H_2_S metabolism contribute to cognitive impairments and neurodegenerative disorders, underscoring the potential therapeutic value of targeting H_2_S-mediated pathways. Although the precise mechanisms of H_2_S-induced changes in synaptic strength remain elusive, a growing body of evidence positions H_2_S as a significant regulator of memory formation processes. This review calls for more rigorous exploration into the molecular underpinnings of H_2_S in synaptic plasticity, paving the way for novel pharmacological interventions in cognitive dysfunction.

## 1. Introduction

Hydrogen sulfide (H_2_S) has long been recognized for its malodorous quality and toxicity at high concentrations, often linked to industrial hazards and volcanic emissions [1]. Despite these negative associations, the discovery of H_2_S as an endogenous signaling molecule has redefined its status in biological systems [2,3,4]. H_2_S stands out for its wide-ranging influence on multiple physiological and pathophysiological processes within the broader framework of gasotransmitters, including nitric oxide and carbon monoxide [5,6]. These include vasodilation, inflammation, cellular metabolism, or neural function [7,8,9,10,11,12,13].

Recent studies have established that H_2_S is far more than a mere byproduct of sulfur metabolism; it can diffuse throughout neural tissues and exert its effects partly through post-translational modifications—persulfidation—with profound implications for synaptic function [14]. Over the past two decades, studies have established that specific enzymes, including cystathionine β-synthase (CBS) and cystathionine γ-lyase (CSE), generate H_2_S in brain cells [15]. H_2_S and polysulfides (H_2_S_n_) [16] are also produced by 3-mercaptopyruvate sulfurtransferase (3-MST) [17]. These findings point to a regulated, rather than merely accidental, presence of H_2_S in the nervous system [18,19,20].

Synaptic plasticity—encompassing long-term potentiation (LTP) and long-term depression (LTD)—underlies cognitive processes such as learning and memory [21]. NMDA receptors trigger intracellular calcium influx upon activation by glutamate and relief of its Mg^2+^ block, triggering cascades and leading to synaptic strengthening or weakening [22].

A growing consensus holds that H_2_S can modulate NMDA receptor function [23,24,25], although the precise molecular mechanisms are not yet fully resolved. Some studies suggest that H_2_S interacts directly with receptor subunits, while others propose that its effects are mediated through alterations in intracellular signaling molecules, including redox-sensitive kinases and second messengers [26]. These explanations reflect the complexity of H_2_S biology and the methodological variations in studying H_2_S in vitro and in vivo [27]. Despite these unresolved details, the capacity of H_2_S to fine-tune NMDA receptor activity aligns with a broader network of established H_2_S actions on neuronal excitability [28,29].

H_2_S and H_2_S_n_ (transient forms) appear to influence neurotransmission beyond the NMDA receptor, particularly in releasing excitatory and inhibitory neurotransmitters such as glutamate and gamma-aminobutyric acid (GABA) [30]. While some data firmly indicate that physiologically relevant H_2_S concentrations can enhance neurotransmitter release and promote synaptic potentiation [31], other studies suggest that higher levels may be neurotoxic or inhibit synaptic function [2,32]. The contrasting findings underscore a possible “Janus-faced” nature of H_2_S [33], where it can be either supportive or detrimental depending on local concentrations, cell type, and the overall metabolic or redox environment [34]. These observations are broadly accepted within the field, although the exact concentration thresholds and mechanistic details remain under investigation [35].

H_2_S biology is dependent on redox-sensitive enzymatic pathways for both synthesis and degradation [36,37]. The levels of H_2_S in neuronal tissue hinge on substrate availability, pH, and the post-translational modification of H_2_S-producing enzymes. Mitochondrial pathways also contribute to H_2_S consumption, linking its homeostasis to the oxidative status of the cell [38,39]. Emerging hypotheses propose that neurons may up- or downregulate H_2_S production in response to shifts in metabolic demand [40,41].

Despite the body of work pointing to a pivotal role for H_2_S in modulating learning and memory through synaptic plasticity, numerous questions remain. For instance, the extent to which H_2_S exerts direct versus indirect influences on NMDA receptor subunits is an ongoing debate. Similarly, the dosage and context dependence of the effects of H_2_S on excitatory or inhibitory neurotransmitter release are not entirely understood, and many contradictory data exist. This leads to speculation about how tightly H_2_S levels must be controlled for optimal synaptic plasticity. Disruptions in H_2_S/H_2_S_n_ production or signaling arising from genetic polymorphisms in H_2_S-producing enzymes or pathological changes in redox homeostasis can potentially contribute to neuropsychiatric disorders [42].

This review aims to synthesize the existing knowledge on H_2_S (or the transient form H_2_S_n_)-mediated synaptic plasticity, focusing on established data regarding its influence on NMDA receptor function and neurotransmitter release. By addressing the molecular, cellular, and physiological dimensions of H_2_S signaling, this review seeks to delineate what is conclusively known, what is suggested, and what remains speculative in the quest to understand how H_2_S shapes learning, memory, and broader cognitive processes.

## 2. Multifaceted Roles of H_2_S in Synaptic Plasticity: Mechanisms, Behavioral Correlates, and Disease Implications

### 2.1. Presynaptic and Postsynaptic Modulation and Neurotransmitter Release

Presynaptic modulation is a fundamental side of synaptic communication, shaping how effectively neurons can initiate and sustain neurotransmission [16,43]. H_2_S has emerged as a significant modulator of the presynaptic machinery, altering excitatory and inhibitory neurotransmitter release across various neuronal subtypes [15]. One of the most direct indicators of H_2_S’s presynaptic influence is the increased frequency of miniature excitatory postsynaptic currents (mEPSCs) observed upon acute H_2_S application [30,44,45]. Because mEPSCs reflect the spontaneous fusion of synaptic vesicles at excitatory terminals, a rise in their frequency suggests that H_2_S elevates the probability of vesicular glutamate release [30]. This effect can pivotally impact synaptic strength and information flow, even without action potential-evoked signaling [44].

Multiple lines of evidence point to the sulfhydration of presynaptic proteins as a key mechanism by which H_2_S enhances release probability [46,47]. Critical components of the vesicle fusion machinery, such as soluble N-ethylmaleimide-sensitive factor attachment protein receptor (SNARE) proteins, contain reactive cysteine residues capable of undergoing H_2_S-mediated post-translational modifications [48,49]. H_2_S can stabilize or accelerate the conformational rearrangements required for vesicle fusion through sulfhydration.

Voltage-gated calcium channels (VGCCs) in the presynaptic membrane may also be directly or indirectly influenced by H_2_S, leading to increased calcium influx. Since presynaptic calcium entry is the primary trigger for synaptic vesicle exocytosis, any alteration in VGCC function can have far-reaching consequences for neurotransmitter release [50,51].

Importantly, H_2_S’s presynaptic actions are not confined to excitatory circuits. Low doses of H_2_S can facilitate gamma-aminobutyric acid (GABA) release from specific interneuron populations, thereby modulating inhibitory tones within local circuits [30,52,53].

Conversely, higher H_2_S concentrations or prolonged exposure can yield biphasic effects, where a decline follows the initial facilitation of release in neurotransmitter exocytosis [54]. These complex outcomes emphasize the dynamic and context-dependent nature of H_2_S’s role in presynaptic modulation. Factors such as synaptic identity, the local redox environment, and the specific complement of sulfhydration targets all contribute to the ultimate effect of H_2_S on neurotransmitter release.

Beyond its presynaptic effects, H_2_S exerts critical postsynaptic actions that substantially affect synaptic potentiation and network-level plasticity [35]. One prominent mechanism involves H_2_S-mediated sulfhydration of AMPA receptor subunits, including GluA1 [55]. By modifying cysteine residues on these subunits, H_2_S can enhance receptor stability, alter channel conductivity, and regulate receptor trafficking to or from the postsynaptic density. Such modifications are closely tied to synaptic strength: for instance, increased GluA1 surface expression generally correlates with heightened excitatory postsynaptic currents and facilitated LTP [56].

H_2_S also influences calcium signaling by modulating VGCCs and receptors linked to intracellular calcium release, thus calibrating downstream kinase and phosphatase cascades [49]. Within this network, the mammalian target of the rapamycin/phosphoinositide 3-kinase/protein kinase B (mTOR/PI3K/AKT) axis emerges as a particularly relevant target, where H_2_S-initiated sulfhydration or redox modulation can affect kinase activity or substrate specificity [57]. Fluctuations in this axis lead to altered protein synthesis, dendritic spine morphology shifts, and neuron survival pathway adjustments [43]. Integrating these postsynaptic perspectives highlights that H_2_S is a multifunctional neuromodulator, modulating both the presynaptic machinery and the postsynaptic molecular landscape in ways that ultimately shape learning, memory, and adaptive brain functions.

### 2.2. H_2_S Modulates NMDA Receptor Subunits and Calcium Influx

**Figure 1:**
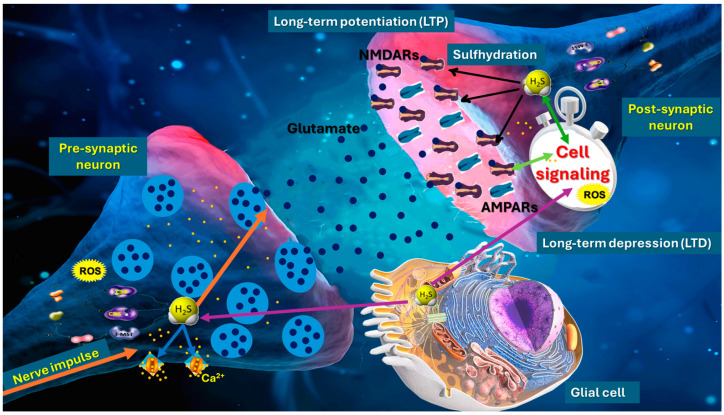
Role of hydrogen sulfide (H_2_S) in synaptic plasticity, particularly in the modulation of long-term potentiation (LTP) and long-term depression (LTD). H_2_S, synthesized by enzymes such as cystathionine β-synthase (CBS), cystathionine γ-lyase (CSE), and 3-mercaptopyruvate sulfurtransferase (3-MST), regulates synaptic transmission through sulfhydration of presynaptic proteins and modulation of calcium channels, enhancing neurotransmitter release. In the postsynaptic neuron, H_2_S modifies NMDA receptor (NMDAR) activity via sulfhydration, promoting calcium influx and activating signaling pathways essential for synaptic strengthening. Astrocytes contribute by providing metabolic support and influencing extracellular H_2_S and neurotransmitter levels. This interplay between H_2_S, ROS, and NMDARs shapes synaptic strength. H_2_S acts as a dual regulator, promoting synaptic plasticity and neuroprotection.

H_2_S has gained recognition as a powerful neuromodulator that influences a range of ionotropic [58] and metabotropic receptors [59]. Among its most studied targets are NMDA receptors (NMDARs), which play a pivotal role in excitatory neurotransmission and synaptic plasticity (Figure 1). NMDARs are heterotetrameric complexes typically composed of two GluN1 subunits and a combination of GluN2 (A–D) subunits, although the exact subunit composition can vary among brain regions and developmental stages [60]. Exogenous H_2_S donors in neuronal preparations frequently enhance NMDAR-mediated currents, indicating an excitatory shift in synaptic transmission [61]. Conversely, the pharmacological blockade or genetic reduction of H_2_S-producing enzymes often attenuates these currents, indicating a requirement for endogenous H_2_S [62,63].

One of the mechanisms by which H_2_S interacts with NMDARs has been partly attributed to sulfhydration, a post-translational modification that targets reactive cysteine residues [64,65,66,67,68]. This modification can induce subtle structural rearrangements that facilitate channel opening and potentially stabilize conductive states, enhancing ion permeability. Neuronal calcium imaging studies corroborate these findings, showing that treatment with H_2_S donors increases intracellular calcium signals [69] in response to glutamate or NMDA [70,71]. These amplified calcium transients, in turn, are known to activate downstream protein kinases, including calmodulin-dependent protein kinases (CaMKs), which are well-recognized effectors in synaptic strengthening [72]. Notably, however, the effects of H_2_S on NMDARs are not universally excitatory; some research indicates that under certain conditions—especially at higher H_2_S concentrations—excessive sulfhydration or redox alterations might reduce receptor activity, signifying a tightly regulated, context-dependent modulatory profile [73,74].

Delving deeper into the molecular underpinnings, H_2_S’s interaction with NMDAR subunits appears closely tied to the receptors’ redox sensitivity. The NMDAR is known to harbor redox-sensitive sites on its extracellular domains, and its function can be modulated by reducing or oxidizing agents [75,76,77]. In this context, H_2_S often acts as a reducing agent, maintaining a more favorable redox potential that prevents the formation of disulfide bonds capable of constraining receptor function. By preserving or creating free thiol groups, H_2_S may keep NMDARs in a conformation more readily activated by glutamate. The specificity of these redox events likely depends on the precise cysteine residues located on different subunits, particularly those of GluN1 and GluN2A/GluN2B, which are most commonly studied in the hippocampus and cortex [7,68,78].

Another critical point involves the temporal dynamics of H_2_S action. Because H_2_S is gaseous and can diffuse rapidly through membranes, its local concentration in the synaptic cleft can quickly fluctuate. Enzymes such as CBS and CSE, responsible for H_2_S production in neurons and glial cells, may thus tailor the timing of H_2_S release to specific physiological events, including periods of high synaptic activity. This synchronized release could enable a tight coupling between NMDAR activation and H_2_S-mediated facilitation [79,80,81].

### 2.3. Effects on Long-Term Potentiation and Long-Term Depression

Parallel to its direct interaction with the NMDA receptor, H_2_S has been involved in shaping the durability and strength of synaptic changes, particularly long-term potentiation (LTP) and long-term depression (LTD) [82], the most intensively studied forms of synaptic plasticity, serving as fundamental mechanisms that underlie learning, memory, and adaptive information processing in the brain [83].

Applying H_2_S donors such as sodium hydrosulfide (NaHS) in hippocampal slice experiments can enhance LTP induction in response to high-frequency stimulation, suggesting a facilitative role in synaptic strengthening [84]. Electrophysiological recordings often show that treated slices display a heightened initial slope of excitatory postsynaptic potentials, reflecting an augmented postsynaptic responsiveness [85]. In contrast, some studies document that interfering with endogenous H_2_S production via enzyme inhibitors diminishes LTP magnitude [86]. Mechanistically, these effects appear closely tied to the modulation of calcium-permeable channels, receptor phosphorylation states, and second messenger cascades. The ability of H_2_S to enhance or suppress LTP/LTD appears intimately tied to its capacity to regulate intracellular signaling cascades, receptor phosphorylation states, and calcium channel dynamics. Thus, while H_2_S has garnered attention for its immediate receptor-level effects, it is equally significant that it modulates enduring changes in synaptic transmission [44,45].

Not all studies show consistent LTP facilitation; in certain experimental conditions, high concentrations of H_2_S donors have been reported to reduce potentiation or even paradoxically induce synaptic depression. This discrepancy underscores the importance of examining H_2_S’s concentration, timing of application, and the local redox environment in determining its net effect on synaptic modifications [87].

Synaptic plasticity relies on the coordinated regulation of signaling molecules such as protein kinase A (PKA), mitogen-activated protein kinases (MAPKs), and CaMKII. These enzymes modulate synaptic strength by phosphorylating receptor subunits (e.g., GluA1 of AMPA receptors) and other postsynaptic density proteins, leading to changes in receptor trafficking and synaptic morphology. Current evidence suggests that H_2_S can adjust the activity of these kinases, either directly through sulfhydration of catalytic or regulatory cysteine residues or indirectly by altering the local redox environment necessary for kinase activation [61,88,89,90].

Additionally, H_2_S may influence phosphatases such as calcineurin (PP2B), which counterbalance kinase activity to maintain synaptic homeostasis. By fine-tuning the balance between phosphorylation and dephosphorylation, H_2_S can tilt synapses toward potentiation when conditions are conducive. Interestingly, these effects may be highly transient, as H_2_S can rapidly metabolize or diffuse away from the synaptic cleft. This fleeting presence suggests that H_2_S spikes during high-frequency stimulation could act as a permissive signal, priming synapses for LTP, provided that subsequent molecular events (such as AMPA receptor insertion) are adequately triggered [91,92,93].

Behavioral studies in animal models further corroborate the link between H_2_S-mediated potentiation and improved learning and memory tasks. Rodents treated with moderate doses of H_2_S donors often show enhanced performance in maze-based tasks or object recognition paradigms [81]. This outcome is frequently associated with heightened LTP in the hippocampus [94]. Conversely, inhibiting H_2_S synthesis can compromise learning-related synaptic plasticity, hinting that endogenous H_2_S is an integral component of normal cognitive function [95].

These data illustrate how H_2_S’s control over protein phosphorylation and enzymatic cascades contributes to LTP maintenance, rendering it an influential, though complex, regulator of synaptic strength. Despite these complexities, the broader takeaway is that H_2_S does not simply act as a one-size-fits-all potentiation signal.

### 2.4. Redox-Dependent Mechanisms and Sulfhydration of Synaptic Proteins

Accumulating data suggest that the redox properties of H_2_S underlie a significant portion of its modulatory capacity, especially at synapses characterized by high metabolic demand [96]. By acting as both a reducing agent and a sulfhydrating molecule, H_2_S can stabilize or destabilize protein structures integral to synaptic transmission [97]. Proteomic analyses reveal that key synaptic proteins—ion channels, receptors, and vesicle-associated proteins [98]—harbor reactive cysteine residues amenable to modification by H_2_S [99].

A core mechanism by which H_2_S exerts its redox-based influence lies in sulfhydration, a post-translational modification that selectively targets reactive cysteine residues in proteins [47]. Cysteine residues can act as “hotspots” for H_2_S interaction in specific conformational or microenvironmental contexts [100]. Through sulfhydration, the thiol (-SH) group of cysteine is converted into a persulfide (-SSH), which can cause marked alterations in protein structure, stability, and activity [101]. In synaptic environments, where the confluence of ion fluxes and enzyme activity is exceptionally high, these modifications may transiently reshape the functionality of receptors, ion channels, or scaffold proteins. One particularly illustrative example is the sulfhydration of AMPA receptor subunits. Experimental evidence suggests that this modification can enhance the open probability of AMPA channels, thereby amplifying excitatory currents and potentially synergizing with NMDA receptor activation [55]. The net effect is an environment conducive to LTP, a cellular correlate of learning and memory.

Sulfhydration is often transient, with reversal possible through reducing agents or enzymatic pathways. This dynamic quality allows neurons to harness H_2_S as a rapid-response signal. Under intense synaptic activity, local bursts of H_2_S may propagate wave-like changes in protein function. Once baseline activity resumes, reverse modifications or proteolytic turnover can restore proteins to their default states. Hence, sulfhydration emerges as a flexible yet potent means for redox-based fine-tuning synaptic transmission [64].

Alongside sulfhydration, H_2_S exerts broader redox influences on synaptic apparatus. By acting as a reducing agent, H_2_S can neutralize certain reactive oxygen species or at least modulate their local concentrations [102]. This antioxidant-like behavior is particularly evident in neurons with high metabolic rates, such as those in the CA1 region of the hippocampus, where oxidative phosphorylation is robust [103]. When oxidative species accumulate during periods of intense firing or metabolic stress, H_2_S may help preserve the functional integrity of proteins by preventing cysteine oxidation or disulfide bond formation. In this protective role, H_2_S complements traditional antioxidants such as glutathione, collectively maintaining a redox environment favorable to sustained synaptic function.

Nonetheless, the flip side of H_2_S’s redox capacity arises under conditions of severe oxidative stress. Excessive ROS can react with H_2_S to generate alternative sulfur-containing species, which may be more reactive or damaging than H_2_S [104]. These compounds can catalyze oxidative modifications that inactivate critical synaptic enzymes or degrade membrane lipids, undermining neuronal excitability. Moreover, redox imbalances can shift the equilibrium from beneficial sulfhydration to detrimental oxidation states, effectively negating H_2_S’s usual facilitative roles [105]. Hence, the context of the local redox balance is paramount. While moderate H_2_S levels in a relatively reduced environment enhance synaptic performance, the same H_2_S levels in an overtly oxidized setting might contribute to pathological modifications of proteins.

This dualistic nature has far-reaching implications for synaptic plasticity. Positive redox modulation via H_2_S can heighten the efficacy of glutamatergic and GABAergic transmission, sharpen synaptic responsiveness, and stabilize plastic changes [106]. Conversely, if the oxidative burden overwhelms normal homeostatic controls, H_2_S’s redox reactivity might amplify detrimental processes, leading to mitochondrial dysfunction, impaired calcium homeostasis, or even excitotoxicity [107].

### 2.5. Regional Specificity and Network-Level Outcomes

The actions of H_2_S on synaptic plasticity and neuronal excitability have been extensively studied in the hippocampus, reflecting the central importance of this region in learning and memory. However, a broader survey of the nervous system reveals that H_2_S does not exert uniform effects across all brain areas [61]. Instead, distinct anatomical structures exhibit varying susceptibilities to H_2_S, partly stemming from differences in baseline metabolic demands, neuronal circuitry, and local expression patterns of H_2_S-producing enzymes. For instance, while the hippocampus may display pronounced levels of CBS, other regions can rely more heavily on CSE or 3-MST, resulting in unique H_2_S “profiles” that shape how synapses respond to endogenous or exogenous H_2_S [108,109].

Cortical pyramidal neurons, which integrate and process complex inputs from various subcortical and sensory areas [110,111], can exhibit changes in synaptic strength upon H_2_S application. However, the magnitude and direction of these changes may differ from hippocampal neurons. In specific cortical layers, for example, H_2_S could enhance excitatory transmission, whereas in others, it might differentially modulate inhibitory interneurons. This layer-dependent heterogeneity underscores the need to investigate not only the region as a whole but also its micro-architectural organization [112,113].

Investigations in the cerebellum highlight how H_2_S might modulate motor coordination and learning. Purkinje cells, the principal neurons of the cerebellar cortex, receive excitatory inputs from parallel fibers and climbing fibers [114,115,116,117]. Changes in H_2_S levels can influence the plasticity at parallel fiber–Purkinje cell synapses and the overall balance of excitatory/inhibitory signals in cerebellar microcircuits [118]. H_2_S could fine-tune motor learning processes, such as adapting to new tasks or adjusting motor outputs after sensory feedback [119]. These region-specific observations collectively emphasize that H_2_S must be considered within the functional context of each brain structure, as its effects on synaptic transmission—and, by extension, behavior—can be highly specialized [94,120].

A significant determinant of region-specific H_2_S dynamics lies in the differential expression and regulation of the enzymes responsible for its synthesis. CBS, CSE, and 3-MST have distinct tissue distributions, catalytic properties, and regulatory mechanisms [5].

In some areas, such as the hippocampus, CBS may be highly expressed in astrocytes and specific interneuron populations, leading to significant local H_2_S production when these cells are metabolically active [121]. In the cortex, a balance between CBS and CSE might yield lower or more fluctuating levels of H_2_S, potentially resulting in different baseline concentrations or release patterns (Table 1). As neurons and glia become engaged in region-specific tasks, such as encoding spatial memory in the hippocampus or orchestrating fine motor control in the cerebellum, the flux of H_2_S can shift accordingly [122,123,124].

Additionally, local factors such as oxygen tension, redox state, and substrate availability (e.g., cysteine) modulate the activity of H_2_S-producing enzymes. This interplay can create microenvironments in which H_2_S levels deviate even within the same brain region. For example, subregions of the hippocampus (CA1 vs. CA3) or different cortical layers (II/III vs. V) may exhibit unique metabolic profiles that render them more or less prone to H_2_S fluctuations. Consequently, synaptic terminals in high-enzyme or high-substrate zones might experience robust H_2_S signaling during episodes of heightened activity, while neighboring areas remain relatively unaffected [125,126,127].

**Table 1:** Comparative overview of H_2_S’s effects in different brain regions.

Brain Region	H_2_S-Producing Enzymes	Principal Mechanisms	Observed Functional/Behavioral Outcomes	Ref.
**Cortex**	CBS, CSE, and possibly 3-MST	- Modulates excitatory versus inhibitory balance - Alters glutamate/GABA neurotransmission - Influences astroglial clearance of neurotransmitters	- Contributes to cortical plasticity and potentially to stress responsiveness - May interface with nitric oxide (NO) and carbon monoxide (CO)	[110,111,118]
**Hippocampus**	High CBS expression, CSE, and 3-MST	- Fine-tunes NMDA receptor function and presynaptic glutamate release - Facilitates LTP or LTD depending on concentration - Protein sulfhydration	- Enhances spatial learning, memory encoding, and synaptic consolidation - Modulates CA1–CA3 circuit excitability - Protective at moderate levels (neuroprotective); detrimental under high oxidative stress	[95,108,128,129]
**Basal Ganglia** (e.g., the striatum)	Primarily CSE (CBS is relatively lower)	- May influence dopaminergic and GABAergic pathways - Redox regulation of key proteins involved in motor control - Possible interactions with inflammatory processes	- Potential role in modulating motor coordination and reward-related behavior - Dysregulation could impact nigrostriatal pathways, contributing to motor deficits	[52,87]
**Cerebellum**	CBS and CSE in Purkinje cells/glia	- Adjusts synaptic plasticity at parallel fiber–Purkinje cell synapses - Modulates interplay of excitatory (glutamatergic) and inhibitory (GABAergic) inputs to Purkinje neurons	- Contributes to fine-tuning motor coordination and adaptive learning - Helps calibrate sensorimotor integration and error correction - Abnormal H_2_S signaling might exacerbate cerebellar dysfunctions	[114,117]

At the network level, the cumulative impact of H_2_S and H_2_S_n_ on individual synapses can manifest as altered patterns [27] of oscillatory activity or connectivity. Neural oscillations, such as theta (4–8 Hz) and gamma (30–80 Hz) rhythms, are integral to functions such as memory consolidation, sensorimotor integration, and attention [130,131]. Studies using in vitro slice preparations have shown that H_2_S donors can shift the balance between excitation and inhibition in ways that modulate these oscillations [128].

### 2.6. Synergistic and Antagonistic Interactions with Other Signaling Molecules

Several studies have probed the interplay between H_2_S and other neuromodulators or gasotransmitters, revealing a web of synergistic and antagonistic relationships that shape final synaptic responses. One frequently cited example is the crosstalk between H_2_S and nitric oxide (NO), where combined exposure to both gases can either potentiate or suppress NMDA receptor function depending on their relative concentrations and the timing of release [132,133]. Beyond NO, H_2_S also converges with other signaling messengers to shape synaptic outcomes. For instance, a recurring theme in synaptic plasticity is the generation of second messengers such as cyclic AMP (cAMP) and cyclic GMP (cGMP). These molecules activate protein kinases—PKA and PKG, respectively—that phosphorylate key substrates involved in LTP induction, including AMPA receptor subunits and synaptic scaffolding proteins. Recent findings suggest that H_2_S can either amplify or dampen these pathways, depending on concentrations and cellular contexts [26,134].

Carbon monoxide (CO) can also diffuse freely across cell membranes, modulate ion channels, influence second messenger pathways, and participate in post-translational modifications that affect neuronal excitability and synaptic plasticity [135]. One of the primary sources of CO in the brain is the enzymatic degradation of heme by heme oxygenase (HO), which exists in two main isoforms: HO-1, which is often upregulated in response to stress or injury, and HO-2, which is more constitutive and associated with neuronal function. CO can influence neurotransmission through this route by interacting with soluble guanylate cyclase (sGC), thereby modulating cyclic GMP levels and enhancing kinase-mediated phosphorylation events that alter synaptic receptor sensitivity [136]. CO also converges with H_2_S and NO in pathways regulating inflammatory cytokine production, redox balance, and glial reactivity, suggesting a complementary or synergistic role under certain physiological or pathophysiological conditions [137].

Neurotrophic factors represent another critical layer of synergy with H_2_S. Molecules such as brain-derived neurotrophic factor (BDNF) [138] and glial-derived neurotrophic factor (GDNF) [139] are well-known for shaping synaptic growth, maintenance, and plasticity. These factors signal primarily through receptor tyrosine kinases—TrkB for BDNF, for example—initiating cascades that bolster synapse formation, receptor expression, and neuronal survival. Emerging evidence suggests that H_2_S can influence either the expression or secretion of these neurotrophic factors in select brain regions, potentially boosting or impairing synaptic plasticity depending on context [140].

In parallel, H_2_S could also modulate the intracellular signaling downstream of BDNF receptors, including pathways such as MAPK/ERK [141] and PI3K/Akt/mTOR [142,143]. By selectively sulfhydrating regulatory cysteines on enzymes within these pathways, H_2_S may change the phosphorylation states of target proteins, amplifying or attenuating BDNF’s usual effects on synaptic plasticity [144].

Another crucial interaction occurs between H_2_S and metabolic signals that govern neuronal energy states. The brain is an energetically demanding organ, and neurons finely tune their activity according to the availability of glucose, oxygen, and other metabolic substrates. AMP-activated protein kinase (AMPK) is a central energy sensor that upregulates catabolic pathways when ATP levels drop [145]. Preliminary data suggest that H_2_S can feed into this energy-sensing machinery, modulating AMPK activity directly through sulfhydration or indirectly by influencing mitochondrial function [57,127].

The multi-layered nature of H_2_S’s crosstalk extends to inflammatory mediators and immune components within the central nervous system. Microglial cells, the brain’s resident immune cells, release cytokines and other signaling molecules upon activation. While acute inflammatory responses can promote tissue repair and synaptic remodeling, chronic inflammation is often deleterious, contributing to synaptic dysfunction and neurodegeneration. H_2_S, in specific contexts, has been described as an anti-inflammatory agent, capable of attenuating microglial activation or reducing the production of pro-inflammatory cytokines such as TNF-α and IL-1β [19].

**Figure 2:**
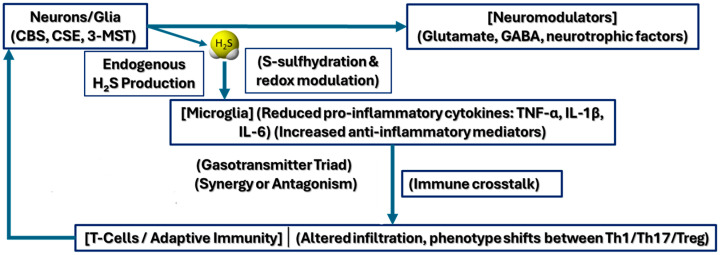
Mechanistic interplay of H_2_S in synaptic and neuroinflammatory pathways. This schematic depicts how H_2_S, generated in neurons and glial cells via cystathionine β-synthase (CBS), cystathionine γ-lyase (CSE), or 3-mercaptopyruvate sulfurtransferase (3-MST), modulates both presynaptic and postsynaptic dynamics in concert with neuroinflammatory processes.

Beyond microglia, H_2_S also modulates adaptive immunity (Figure 2) in autoimmune settings, as demonstrated in multiple sclerosis (MS) models, where T-cell infiltration and activation in the central nervous system (*CNS*) are key drivers of demyelination [61]. H_2_S-mediated redox alterations can influence T-cell trafficking, skew T-cell lineage commitment (e.g., Th17 vs. regulatory T cells), and modify the blood–brain barrier’s permeability to immune cells. By tempering excessive neuroinflammation while supporting critical housekeeping and repair processes [15], H_2_S exerts both direct and indirect influences on synaptic homeostasis. This dual role reflects the complexity of H_2_S signaling in diseased states, where small shifts in local H_2_S levels or redox balance may tip the scale between protective or detrimental immune responses, offering a potential therapeutic avenue for disorders marked by chronic neuroinflammation such as MS [146].

Additionally, crosstalk with astrocytes is key, as these glial cells help regulate extracellular glutamate levels and maintain synaptic homeostasis. H_2_S’s modulation of astrocytic function could indirectly modulate excitotoxic outcomes during inflammation, either supporting the clearance of glutamate or, under pathological conditions, failing to prevent glutamate spillover and neuronal damage [147,148].

**Figure 3:**
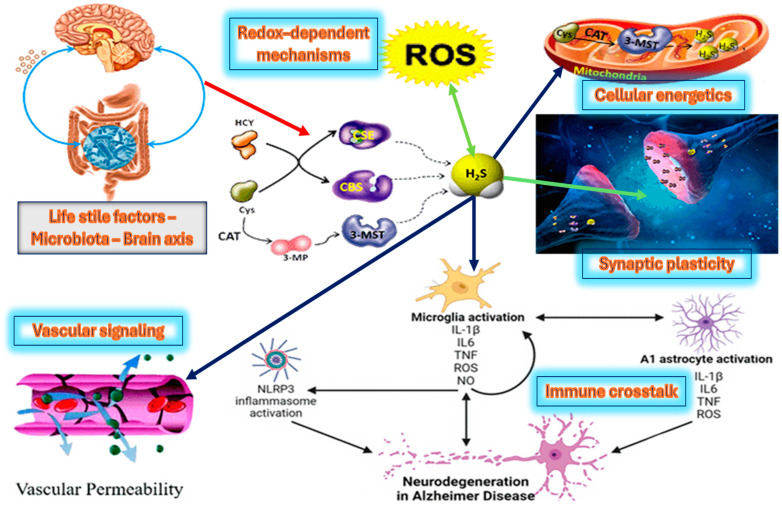
H_2_S as a key regulator of neurobiology and neurodegeneration, showing how lifestyle factors (e.g., diet, physical activity, and microbiota composition) can modulate H_2_S-generating enzymes. H_2_S also boosts synaptic plasticity, supporting processes such as long-term potentiation. By counteracting NOD-like receptor family pyrin domain-containing 3 (NLRP3) inflammasome activation, H_2_S can attenuate the local production of IL-1β and help prevent the sustained microglial overactivation that drives neurodegenerative progression.

Synergy and antagonism between H_2_S and inflammatory mediators operate on multiple timescales. Acutely, H_2_S might quell excessive immune activity and support neuronal health; chronically, inflammatory conditions could subvert H_2_S’s beneficial roles, turning it into a factor that amplifies neurodegenerative processes (Figure 3). The nucleotide-binding oligomerization domain-like receptor protein 3 (NLRP3) inflammasome is a central mediator of neuroinflammation. H_2_S appears to exert regulatory control by inhibiting NLRP3 activation, thereby preserving synaptic integrity and supporting neuroprotection in the context of chronic brain pathologies [129].

### 2.7. Behavioral Correlates of H_2_S-Mediated Synaptic Plasticity

Translating the synaptic actions of H_2_S to behavior, several animal studies connect pharmacological or genetic manipulation of H_2_S levels with changes in learning and memory tasks. For instance, rodents receiving intracerebral H_2_S donors often show improved performance in spatial navigation or fear conditioning paradigms, consistent with enhanced synaptic plasticity in hippocampal circuits [149]. Notably, the effects on behavior often hinge on the specific dosage and route of administration of H_2_S donors, as well as the animal’s baseline physiological state (e.g., age, stress level, or presence of neuropathology). These behavioral outcomes, while preliminary in many cases, lend further support to the view that H_2_S modulation of synaptic plasticity has functionally significant consequences extending from the molecular scale to complex cognitive tasks.

One of the prominent behavioral domains in which H_2_S has been implicated is emotional processing, particularly regarding anxiety and depression-like phenotypes in rodents [150]. Experiments involving elevated plus mazes, light–dark box tests, and forced swim tests reveal that exogenous H_2_S administration can yield mild anxiolytic or antidepressant-like effects [151]. For example, rodents treated with moderate doses of H_2_S donors sometimes spend more time exploring open arms in elevated plus mazes, suggesting reduced anxiety [129].

### 2.8. Implications for Disease Models and Therapeutic Potential

Considerable attention has been paid to the role of H_2_S in disease models characterized by synaptic dysfunction, such as Alzheimer’s disease, Parkinson’s disease, Huntington’s disease, and various forms of dementia [152,153,154,155]. Studies show that in some models of neurodegeneration, boosting H_2_S levels via targeted donors can partially rescue deficits in LTP and memory performance, potentially by alleviating oxidative stress, preserving mitochondrial function, or regulating pathological protein aggregation [156]. Conversely, in models where excitotoxicity drives synaptic loss, excessive H_2_S could intensify damage if it further elevates glutamatergic transmission beyond tolerable thresholds [157]. Consequently, the therapeutic window for H_2_S appears narrow, underscoring the importance of careful dosing and delivery strategies. Intriguing work on novel H_2_S-releasing compounds, prodrugs, and nanoparticle-based delivery systems suggests that a more precise manipulation of H_2_S in the brain is achievable [158]. These emerging technologies have fueled optimism that harnessing H_2_S to restore or enhance synaptic plasticity could become a viable adjunct treatment for neurodegenerative and neuropsychiatric disorders. Nonetheless, translating these findings into clinical practice will require resolving ongoing controversies about dosage, timing, and off-target effects and advancing our understanding of how H_2_S integrates with other pathogenic processes [61].

## 3. Discussion

This review underlines the multifaceted roles of H_2_S in modulating synaptic plasticity, offering insights into the molecular intricacies and the broader physiological significance of this gaseous transmitter. H_2_S can influence NMDA receptor function, alter LTP and LTD, and modulate presynaptic neurotransmitter release [159]. Therefore, H_2_S is a crucial regulator of neuronal signaling [160].

Neurons routinely experience shifts in metabolic demands and oxidative status, especially during periods of high synaptic activity. Thus, H_2_S-based modifications might serve as a real-time sensor, linking the cell’s metabolic health to synaptic tuning. When conditions are favorable, H_2_S could promote plasticity by stabilizing or enhancing receptor function [161]. Conversely, alterations in H_2_S metabolism could signal a need to recalibrate synaptic output in the face of neuronal stress [7].

Translating H_2_S-mediated synaptic modifications into observable behavioral changes forms a critical bridge between basic neurochemistry and cognitive function. Studies demonstrating that pharmacological manipulation of H_2_S can alter learning and memory tasks in animal models strongly suggest a causal relationship [56]. Yet, the behavioral consequences of H_2_S application are not universally positive: specific doses or modes of administration can impair learning, indicating that too much or too little H_2_S may disrupt cognitive processes.

While clinical research on H_2_S is still in its relative infancy, initial human trials or compassionate-use cases could soon offer insights into practical dosing regimens, side effect profiles, and patient selection criteria. If such trials are to succeed, they must address not only safety but also the reproducibility of H_2_S’s neurological effects [162]. Despite their utility, animal models cannot fully replicate the complexity and variability of human neurodegeneration. Differences in lifespan, genetics, immune responses, and environmental exposures can all affect whether H_2_S exerts a protective or deleterious influence in individual patients. Consequently, large-scale clinical studies may need adaptive designs that pivot in response to real-time biomarker data, adjusting H_2_S dosing or targeting strategies based on each patient’s evolving pathology [163].

One lesson emerging from these complexities is the necessity of a “systems approach”. Rather than examining H_2_S in isolation, future studies should incorporate broader assessments of neuronal network activity, metabolic parameters, and immune status. Such integrative methodologies could reveal masked patterns when focusing on a single aspect of synaptic physiology. Additionally, computational modeling may help predict how H_2_S levels fluctuate under varying network demands, providing insights into its potential role in maintaining homeostatic plasticity.

“H_2_S supplementation” in a preventative context raises its own scientific and ethical questions. Without a clear biomarker-based threshold of “healthy H_2_S”, any such strategy risks being haphazard [164]. Some individuals may already produce ample H_2_S and see little benefit from supplementation, while others may harbor genetic variants that hinder typical H_2_S enzymatic pathways. Thus, personalized medicine paradigms incorporating genetic testing or metabolomic profiling would be essential for determining who might benefit from prophylactic measures. Additionally, implementing broad public health interventions that promote H_2_S-boosting diets or supplements would require meticulous evidence that these strategies are safe and effective for the general population, not just high-risk groups.

Regarding limitations, most current models still rely on exogenous H_2_S donors that may not accurately replicate endogenous release kinetics. Furthermore, the concentration range for physiological versus pathological H_2_S levels remains debatable. The local microenvironments in which H_2_S acts can differ significantly from those measured in bulk tissue assays. Overcoming these limitations will likely require novel experimental designs integrating new detection methods, genetically encoded sensors, and region-specific manipulations of H_2_S production.

## 4. Conclusions

H_2_S has evolved from a gaseous toxic molecule into a recognized neuromodulator with profound implications for synaptic plasticity, learning, and memory. Through direct interactions with NMDA receptors and the indirect modulation of presynaptic release mechanisms, H_2_S shapes the strength and longevity of synaptic connections. Its effects, however, are highly dependent on local concentrations, enzymatic production, and redox conditions, underscoring a delicate balance in which H_2_S can either bolster neuronal communication or contribute to excitotoxic and oxidative stress pathways.

Future research will benefit from integrating high-resolution imaging techniques, genetically encoded sensors, and region-specific manipulations of H_2_S enzymes to pinpoint how this gas shapes plasticity under different physiological and pathological conditions.

Moving forward, an essential step lies in refining real-time H_2_S detection techniques that accurately reflect dynamic changes in living neural tissue. Fluorescent probes and genetically encoded biosensors under development can offer unprecedented insight into how endogenous H_2_S levels fluctuate during distinct physiological or pathological states, thereby guiding dose–response relationships for therapeutic interventions.

In tandem with more nuanced measurement tools, the design of disease-specific H_2_S donor compounds optimized for release kinetics, tissue targeting, and dosage thresholds could unlock tailored treatments for disorders ranging from Alzheimer’s disease to multiple sclerosis.

Equally promising is the prospect of using H_2_S as a biomarker. If validated through large-scale clinical studies, alterations in circulating or cerebrospinal fluid H_2_S concentrations may serve as early indicators of neurodegenerative progression or exacerbation, allowing timely, targeted interventions.

Collectively, these advances have the potential to transform our grasp of H_2_S biology from a largely correlational perspective into one capable of precise therapeutic exploitation, laying the groundwork for next-generation interventions in neurological health.

## Data Availability

The authors created the figure enclosed in the manuscript using Canava version Canva Setup 1.106.0, Photoshop version 26.5, Microsoft^®^ PowerPoint^®^ 2021 MSO (version 2502 build 16.0.18526.20168).
